# Reciprocal Repression between Sox3 and Snail Transcription Factors Defines Embryonic Territories at Gastrulation

**DOI:** 10.1016/j.devcel.2011.07.005

**Published:** 2011-09-13

**Authors:** Hervé Acloque, Oscar H. Ocaña, Ander Matheu, Karine Rizzoti, Clare Wise, Robin Lovell-Badge, M. Angela Nieto

**Affiliations:** 1 Instituto de Neurociencias, CSIC-UMH, San Juan de Alicante 03550, Spain; 2 National Institute for Medical Research, London NW7 1AA, UK

## Abstract

In developing amniote embryos, the first epithelial-to-mesenchymal transition (EMT) occurs at gastrulation, when a subset of epiblast cells moves to the primitive streak and undergoes EMT to internalize and generate the mesoderm and the endoderm. We show that in the chick embryo this decision to internalize is mediated by reciprocal transcriptional repression of Snail2 and Sox3 factors. We also show that the relationship between Sox3 and Snail is conserved in the mouse embryo and in human cancer cells. In the embryo, Snail-expressing cells ingress at the primitive streak, whereas Sox3-positive cells, which are unable to ingress, ensure the formation of ectodermal derivatives. Thus, the subdivision of the early embryo into the two main territories, ectodermal and mesendodermal, is regulated by changes in cell behavior mediated by the antagonistic relationship between Sox3 and Snail transcription factors.

## Graphical abstract

**Graphical Abstract:**

**Figure:**
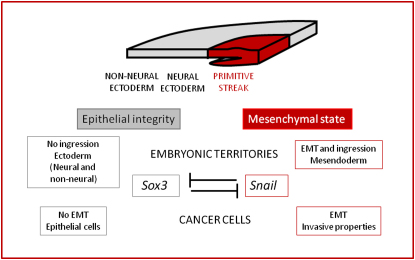

**Highlights:**

► Snail and Sox3 are reciprocal direct transcriptional repressors ► Snail/Sox3 reciprocal repression defines ectodermal versus mesendodermal territories ► Snail2 induces cell delamination without inducing mesodermal or endodermal fates ► The Snail/Sox3 relationship is conserved in mouse embryos and human cancer cells

## Introduction

The shaping of the early embryo involves the conversion of a single layer of ectodermal cells (the epiblast) into a multilayered structure. This complex biological process starts at gastrulation, when a subset of the initial epiblast cells moves inside the embryo to become mesoderm and endoderm (Stern, 2004). In amniotes, gastrulation occurs where Nodal signaling is strongest (Brennan et al., 2001; Bertocchini and Stern, 2002). Initially, in the chick embryo, cells accumulate at the posterior part through epithelial cell intercalation (Voiculescu et al., 2007) in a region now devoid of the hypoblast, a lower layer that inhibits Nodal signaling (Bertocchini and Stern, 2002). This accumulation results in the formation of a midline linear structure called the primitive streak, from which the presumptive mesendodermal cells ingress upon undergoing an epithelial-to-mesenchymal transition (EMT). EMT involves a dramatic change in cell morphology and behavior that allows cells to break the basal lamina, internalize, and start migrating toward their destinations (Harrisson et al., 1991; Acloque et al., 2009). Cells that remain in the epiblast keep their epithelial character and will contribute to the ectodermal derivatives, namely the epidermis, the ectodermal placodes, and the anterior central nervous system (CNS) (Fraser and Stern, 2004). Indeed, much of the CNS will develop from a subset of the noningressing cells later specified as neural precursors. Therefore, it is crucial to identify not only those factors that induce cell ingression at gastrulation but also those that prevent it, because protection from undergoing EMT is necessary to ensure the formation of ectodermal derivatives. Indeed, previous studies have shown that committed neural progenitor cells at the anterior part of the primitive streak are protected from signals that induce internalization. The zinc finger transcription factor Churchill and its target SIP-1 are required to stop ingression movements through the anterior primitive streak (Sheng et al., 2003). This safeguard mechanism operates at stages of neural induction and onset of Churchill expression (from stage HH4+; Hamburger and Hamilton, 1951), but ingression starts at least as early as stage HH2 (Stern and Canning, 1990), suggesting that a different mechanism must exist to protect early ectodermal cells from the EMT inducers.

Among the key factors that induce EMT at gastrulation and that are conserved during evolution are the members of the Snail family (Barrallo-Gimeno and Nieto, 2005). They are fundamental for EMT at gastrulation in all species analyzed and for additional developmental EMT processes (Acloque et al., 2009). We previously found that Snail2 (Slug) downregulation prevented migration from the primitive streak in the chick embryo (Nieto et al., 1994), and here we show that Snail2 is sufficient to induce ectopic delamination in otherwise noningressing epiblast cells, confirming that these cells need to be protected from Snail2 expression and subsequent ingression. Therefore, we set out to identify factors that might prevent Snail expression at early gastrulation, as candidates to play an important role in protecting epiblast cells from undergoing EMT. We show that *Sox3* and *Snail2* are expressed in complementary domains early during gastrulation; gain- and loss-of-function studies reveal that these factors antagonize each other to regulate cell ingression. We show that Sox3-Snail antagonism is implemented through direct reciprocal transcriptional repression, a relationship that seems to be conserved in the mouse embryo and in tumor cell lines, where they also regulate epithelial versus mesenchymal and invasive properties. Together, our results show that Snail-Sox3 cross-repression regulates cell ingression at gastrulation in amniotes and suggest that this antagonistic relationship may also have important implications in cancer.

## Results

### Snail2 Induces Ectopic Cell Delamination in the Ectoderm of the Early Chick Gastrula

**Figure 1:**
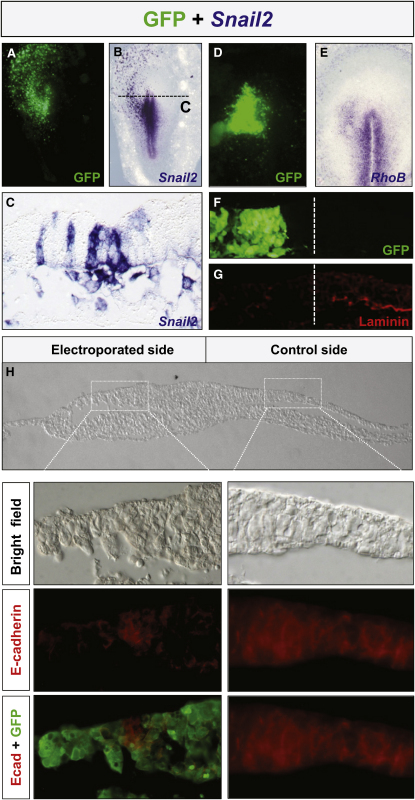
Snail2 Expression in the Epiblast Induces Ectopic EMT and Cell Delamination (A–C) Chick embryos were coelectroporated with a vector encoding GFP and another containing the coding region of *Snail2*. The embryos were subsequently allowed to develop for 15 hr. Endogenous *Snail2* expression at the primitive streak and ectopic expression induced by electroporation could be observed in a dorsal view (B) and in a transverse section through the epiblast (C). Observe the delamination of cells from the epiblast. Dotted line in (B) indicates the level of the section shown in (C). Electroporation of a control empty vector (pCX) or that containing GFP failed to induce cell delamination (not shown). (D–H) Snail2 electroporation was accompanied by the induction of *RhoB* expression, a known Snail2 downstream target (D and E), cell delamination, and disruption of the basement membrane as assessed by the absence of laminin staining (F and G) and by the repression of E-cadherin expression in the electroporated cells (H).

We had previously shown that Snail2 knockdown in the early chick embryo prevents cell ingression at the primitive streak and neural crest delamination from the neural tube (Nieto et al., 1994). To check whether Snail2 is sufficient to trigger EMT and cell delamination, we ectopically expressed the coding region of Snail2 by electroporation of the chick blastoderm at stage 2 (Hamburger and Hamilton, 1951) in the ectodermal region corresponding to the prospective neural plate (see Experimental Procedures). Snail2 ectopic expression induces cell delamination (Figures 1A–1C), suggesting that Snail2 is sufficient to trigger EMT in a territory that normally keeps its epithelial integrity at gastrulation. To confirm that this induced delamination is due to the activation of a full EMT program, we examined the expression of previously described Snail2 target genes known to be necessary for cell delamination, such as the small GTPase *RhoB* (del Barrio and Nieto, 2002) and *E-cadherin* (Cano et al., 2000). As shown in Figures 1D and 1E, Snail2 induces ectopic expression of *RhoB*, together with the disruption of basal lamina, as assessed by the loss of laminin expression (Figures 1F and 1G). Figure 1H shows that E-cadherin is downregulated in electroporated cells that undergo EMT, and cells can be seen delaminating from the epiblast (compare the electroporated and the control sides). These data indicate that Snail2 is sufficient to trigger EMT and cell delamination from the chick epiblast, suggesting that the latter should be protected from Snail expression in the early embryo.

### Snail2 and Sox3 Expression Domains Delineate Ingressing versus Noningressing Embryonic Cell Populations

**Figure 2:**
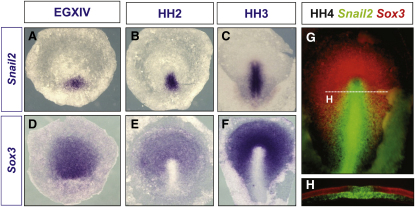
*Snail2* and *Sox3* Genes Are Complementarily Expressed in the Chick Gastrula (A–C) *Snail2* expression starts at the posterior part of the blastula at stage EGXIV (A), and its expression is associated with the primitive streak as it progresses in the gastrulating embryo (B and C). (D–F) *Sox3* expression is detected in the epiblast and it gets progressively downregulated at the primitive streak as it forms. (G and H) Double labeling for *Snail2* (green) and *Sox3* (red) transcripts at the full primitive streak stage (HH4) shows the complementary expression pattern between *Snail2* and *Sox3*. A transverse vibratome section taken at the level shown by the dotted line in (G) allows a better assessment of the mutually exclusive expression pattern and the continued *Snail2* expression in migratory mesendodermal cells emanating from the streak (H).

**Figure 3:**
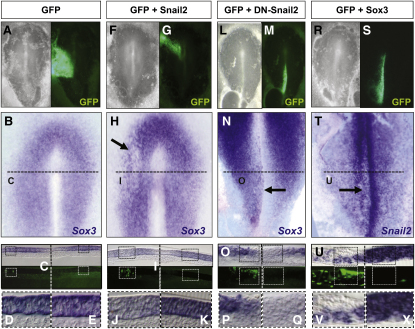
Snail2 and Sox3 Behave as Mutual Transcriptional Repressors in the Chick Embryo (A–E) Control embryo electroporated with a vector encoding GFP (A) shows the normal pattern of *Sox3* expression in a dorsal view (B) and in a transverse section (C). (D) and (E) are high power images of the areas demarcated in (C). (F–K) Similar images taken from an embryo electroporated with *Snail2* in the anterior epiblast shows the repression of *Sox3* expression in the electroporated region (arrow in H and sections in I). High power images comparing the electroporated with the control side confirm *Sox3* downregulation (J and K). (L–Q) Electroporation of a dominant-negative form of Snail2 (DN-Snail2) in the primitive streak extended *Sox3* expression to the embryonic midline (arrow in N), as better assessed in the images shown in (O–Q). (R–X) Ectopic *Sox3* expression by electroporation at the primitive streak inhibited *Snail2* expression (black arrow in T and a section at different magnifications in (U–X).

Because the process of cell ingression has to be tightly regulated to maintain the balance between ectodermal (noningressing) and mesendodermal (ingressing) progenitors, Snail2 is restricted around the primitive streak, where cells are internalized, from the first stages of streak formation (Nieto et al., 1994; Figures 2A–2C). To search for genes functionally equivalent to Churchill but at the early primitive streak stages, we focused on the *Sox3* gene for several reasons: (1) it is highly expressed at epiblast of early-stage embryos, and its expression disappears in the territory surrounding the primitive streak (Rex et al., 1997; Figures 2D–2F); (2) *Sox3* and *Snail2* show mutually exclusive expression patterns (Figures 2G and 2H); and (3) *Sox2*, a closely related gene, has been shown to prevent the induction of *Snail2* by BMP in the dorsal neural fold (Wakamatsu et al., 2004), but it is not expressed at the early gastrulation stages in chick. Therefore, we decided to manipulate either *Snail2* or *Sox3* expression in the chick blastoderm by electroporation and assess the effects on their respective expression. We first induced ectopic expression of *Snail2* by electroporating in regions such as the anterior epiblast. We found that *Sox3* expression was repressed in 75% of the embryos (Figures 3F–3H; black arrow in H; n = 16), which is best seen in transverse sections (Figures 3I–3K). *GFP* electroporation alone does not have the same effect (Figures 3A–3E; n = 13). We then electroporated a Snail2 dominant-negative form lacking the transactivation domain (Morales et al., 2007; DN-Snail2). This led to an extension of *Sox3* expression to the primitive streak up to the midline, a region that normally expresses *Snail2* (Figures 3L–3Q; black arrow in N; 53%, n = 17). Conversely, ectopic Sox3 expression at the primitive streak strongly represses endogenous *Snail2* expression (Figures 3R–3X; 74%; n = 19), indicating that Sox3 and Snail2 act in a mutually antagonistic way.

**Figure 4:**
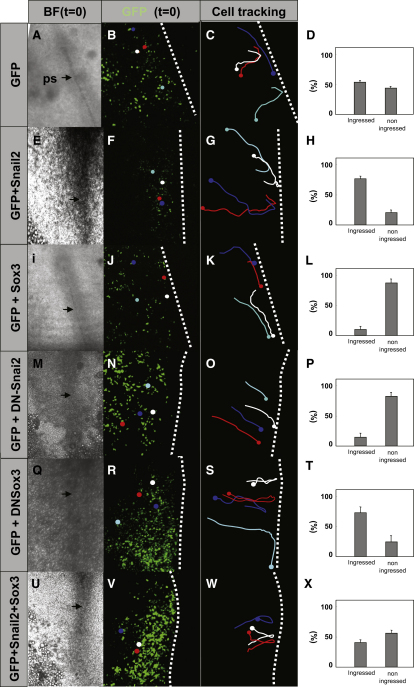
Sox3 Overexpression or Snail2 Inhibition Block Ingression at the Primitive Streak (A–X) Time-lapse confocal analysis of cell movements in cultured embryos electroporated with GFP alone (A–D), GFP plus Snail2 (E–H), GFP plus Sox3 (I–L), GFP plus a dominant-negative form of Snail2 (M–P), GFP plus a dominant-negative form of Sox3 (Q–T), and GFP, Sox3, and Snail2 (U–X). BF (t = 0) and GFP (t = 0) show transmitted light and fluorescent images obtained 6 hr after electroporation, just before the start of time lapse analyses. Colored dots in (B, F, J, N, R, and V) represent the initial position of cells that were subsequently followed. Dots in (C, G, K, O, S, and W) represent the final position of those cells and the lines their tracks. (D), (H), (L), (P), (T), and (X) show the percentages of ingressing and noningressing cells in each condition. The white dotted lines indicate the relative position of the midline; black arrows and ps indicate primitive streak. Control GFP-expressing embryos showed the normal movements of epiblasts cells toward the primitive streak and subsequent ingression (C). Snail2 overexpression increases the percentage of ingressing cells (E–H). Ectopic Sox3 expression blocked cell ingression through the primitive streak without affecting the convergence movement toward the midline (compare C and D with K and L). A dominant-negative form of Snail2 also blocked cell ingression (compare C and D with O and P), whereas a dominant-negative form of Sox3 increased ingression (compare T with D). Overexpression of both Snail2 and Sox3 could rescue the ingression behavior of epiblast cells (compare C and D with W and X).

Next, we examined whether the observed changes in the expression domains of *Snail2* and *Sox3* correlate with cell behavior. With this aim, we followed cell movements near the primitive streak in embryos electroporated with GFP-containing control vectors or with *GFP* plus different *Snail2* and *Sox3* constructs. Cells expressing GFP converge at the primitive steak, ingress, and migrate away in the mesendoderm as expected for a normal embryo (Figures 4A–4D and Movies S1 and S2 available online). Overexpression of wild-type Snail2 increases the proportion of cells that ingress (Figures 4E–4H and Movies S3 and S4). After ectopic expression of *Sox3*, electroporated cells still converge at the primitive streak but are unable to ingress (Figures 4I–4L and Movies S5 and S6). This observation confirms that Sox3 needs to be downregulated to allow cell ingression at the primitive streak, but does not impair the convergence of epiblastic cells at the primitive streak. If the defect in cell ingression is mediated by the observed *Snail2* downregulation by Sox3 (Figures 3R–3X), preventing Snail2 function should elicit a similar defect. Indeed, overexpression of DN-Snail2 in the primitive streak also inhibits cell ingression (Figures 4M–4P and Movies S7 and S8), which is compatible with the observed activation of Sox3 expression (Figures 3L–3Q). In contrast, loss of Sox3 function by ectopic expression of DN-Sox3 close to the primitive streak favors cell ingression (Figures 4Q–4T and Movies S9 and S10), as also observed after Snail2 overexpression (Figures 4E–4H). On the other hand, overexpression of Snail2 together with Sox3 and GFP in the region of endogenous ingression rescued the phenotype of Sox3 overexpression, as the cell movements observed resembled those in the control embryo (Figures 4U–4X and Movies S11 and S12). Altogether, these results strongly suggest that the decision to ingress at the primitive streak depends on the interactions between Snail2 and Sox3 transcription factors.

### Snail2 and Sox3 Are Reciprocal Direct Transcriptional Repressors

**Figure 5:**
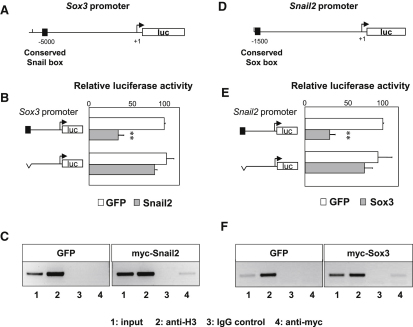
Snail2 and Sox3 Are Direct Mutual Transcriptional Repressors (A) Conserved Snail-binding site in the *Sox3* promoter (see also Figure S1). (B) Snail2 overexpression in cultured embryos repressed the activity of the *Sox3* promoter, whereas a deletion that removes the Snail binding site significantly restores promoter activity. (C) Chromatin immunoprecipitation (ChIP) analysis confirms that Snail2 can directly bind to the *Sox3* promoter. ChIP assays were carried out with anti-myc antibodies on embryo extracts that were previously electroporated with either GFP or myc-Snail2. PCR fragments were obtained after amplification of the indicated promoter region from the input (1), histone H3 positive control (2), IgG negative control (3), and myc immunoprecipitated fractions (4). (D) Conserved sequence for Sox transcription factors binding in the *Snail2* promoter (see also Figure S1). (E) Sox3 overexpression in cultured embryos repressed *Snail2* promoter activity, and the deletion of the Sox-binding site restored most of the promoter activity. (F) Chromatin immunoprecipitation (ChIP) analyses confirmed that Sox3 can directly bind to the *Snail2* promoter. ChIP assays were carried out as described above.

Next, we investigated whether Snail2 and Sox3 can bind to each other's promoter to directly repress expression. We found one Snail-binding site in sequences located 5′ to the *Sox3* coding region, which is conserved in chick, mouse, and human (Figure S1). First, we used a luciferase reporter driven by the 5′ Sox3 sequences (Figure 5A), to test whether Snail2 can modulate the activity of the *Sox3* promoter by coelectroporating *Snail2* with the luciferase construct in the epiblast of preprimitive streak stage embryos (see Experimental Procedures). Snail2 reduced the activity of the *Sox3* promoter construct. This repression is dependent on the presence of the conserved Snail2-binding box (Figure 5B), because electroporation with a construct lacking this box significantly restores activity (Figure 5B). To determine whether Snail2 can bind directly to the Snail box in vivo, we performed Chromatin immunoprecipitation (ChIP) experiments after electroporating embryos with *GFP* and a *myc*-tagged version of *Snail2*. After dissecting the GFP-positive tissues, we carried out ChIP with an anti-myc antibody. A fragment of genomic DNA corresponding to the Snail-box containing region of the *Sox3* promoter was amplified in embryos electroporated with the myc-tagged Snail2 protein (Figure 5C, compare line 4 for GFP and myc-Snail2 experiments). We then performed similar experiments to determine whether Sox3 acts on a similar way on the *Snail2* promoter and found that this is indeed the case (Figures 5D–5F). The conserved Sox DNA binding sequence located in the chick *Snail2* promoter is shown in Figure 5D, and its conservation in other species is shown in Figure S1. Thus, Snail2 and Sox3 can bind directly to each other's promoter in vivo at conserved elements, providing a molecular mechanism for their antagonistic role in the cell decision to ingress at gastrulation. This interaction defines two main territories within the early embryos: the noningressing ectoderm and the ingressing mesendoderm.

### Snail2 Represses Ectodermal Markers without Inducing Mesodermal Fate

**Figure 6:**
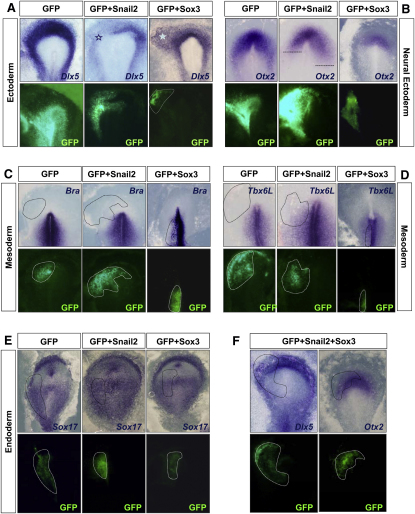
Snail2 Represses Neural and Nonneural Ectodermal Markers without Inducing Mesodermal or Endodermal Fate (A–E) Snail2 or Sox3 were electroporated together with GFP in the epiblast of stage HH2 embryos. Snail2 repressed the expression of *Dlx5*, a nonneural ectodermal marker (A) and *Otx2*, an anterior neural marker (B), whereas Sox3 extended Dlx5 expression (A). Snail2 was unable to induce the expression of mesodermal markers, as assessed by examining the expression of the T-box genes *Brachyury* and *Tbx6L* (C and D) or the endodermal marker *Sox17* (E), whereas Sox3 strongly repressed mesodermal (C and D) but not endodermal markers (E). Overexpression of both Snail2 and Sox3 did not affect the expression of the ectodermal markers *Dlx5* and *Otx2* (F), suggesting that their repression by Snail2 acts through *Sox3* downregulation.

In addition to a strong repression of *Sox3* expression in the epiblast, Snail2 is also able to repress other ectodermal markers, such as the epidermal marker *Dlx5* (Figure 6A; 100%; n = 3) and the neuroectodermal marker *Otx2*, albeit in a limited territory (Figure 6B; 62%; n = 8). This is consistent with the known role of Snail as an epithelial repressor (Nieto, 2002). In contrast, Sox3 induces Dlx5, although it is unable to activate Otx2 expression (Figure 6A; 80%; n = 5; Figure 6B; 100%; n = 11).

We had observed that, in addition to repressing epithelial markers, Snail2 induces delamination of epiblast cells by triggering EMT (Figure 1). We wondered whether this morphological change is accompanied by the induction of mesendodermal fate, because primitive streak cells are *Snail2* positive and give rise to mesoderm and endoderm. However, Snail2 electroporation did not induce the expression of the primitive streak and mesoderm markers *Brachyury* or *Tbx6L* in any of the embryos analyzed (Figures 6C and 6D; n = 5 and n = 8, respectively). Similarly, Snail2 could not induce the expression of the endodermal marker *Sox17* (Figure 6E; 100%; n = 12). In turn, ectopic expression of Sox3 in the primitive streak dramatically reduces the expression of mesodermal markers (Figures 6C and 6D; 100%; n = 8 and n = 9, respectively), as expected from the reduction observed in cell ingression (Figures 4I–4L). Sox3 is unable to induce *Sox17* (Figure 6E; 100%; n = 9), in agreement with the endodermal fate being determined in cells after ingression (Takatori et al., 2010). Altogether, our data suggest that Snail and Sox3 do not behave as mesodermal-endodermal or neural inducers, respectively, but rather as regulators of cell behavior and movement. This is in agreement with the phenotype of *Snail* mutant mouse embryos, which still form mesoderm but are unable to migrate because of a defective EMT at the primitive streak (Carver et al., 2001). It is also compatible with Sox3 being an early neural marker and expressing cells becoming definitive neural only later when they express Sox2 (Linker and Stern, 2004). Furthermore, as expected from the antagonistic relationship between Snail2 and Sox3, the effects of Snail2 on *Dlx5* and *Otx2* expression depend on Sox3 suppression, because overexpression of Snail2 together with Sox3 in the presumptive neural ectoderm rescued their inhibition (Figure 6F; 67%; n = 6).

These results indicate that, even though the antagonistic relationship between Sox3 and Snail2 divides the early embryo in two main territories from which either the ectoderm or the mesendoderm will form, it is cell adhesion and behavior rather than the induction of a change of cell fates that drives the segregation.

### Conservation of the Sox3/Snail Antagonistic Relationship in Mouse Embryos

**Figure 7:**
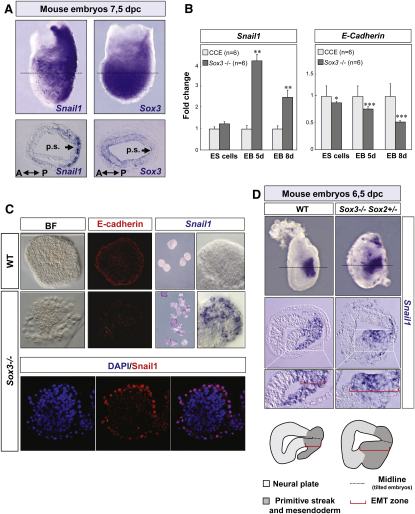
The Antagonistic Relationship between Sox3 and Snail Factors Is Conserved in the Mouse (A) Expression of *Snail1* and *Sox3* in 7,5 d.p.c. mouse embryos. Snail1 is the family member expressed in the primitive streak and early mesendodermal cells in the mouse (Sefton et al., 1998). Note the similarities in the expression patterns between chick and mouse embryos. A mutually exclusive expression pattern is observed in the transverse sections obtained at the level of the primitive streak. (B) *Snail1* expression is increased in embryoid bodies (EB) obtained from Sox3 knockout embryonic stem cells compared to wild-type stem cells (CCE line). Consistent with the increase in Snail1 expression, *E-cadherin* transcripts are downregulated in Sox3 null EBs (T-test statistical analysis). (C) EBs derived from the CCE line are round and smooth, whereas those derived from *Sox3* null ES cells show irregular edges with budding cells delaminating from the EBs, leading to abundant isolated cells in the culture medium (not shown). E-cadherin is strongly downregulated in Sox3^−/−^ compared to wild-type (WT) EBs. Conversely, Snail1 mRNA and protein are detected only in *Sox3*^−/−^ EBs, mainly in cells at the edges. (D) Expression of *Snail1* in WT and *Sox3*^−/−^; *Sox2*^+/−^ mouse embryos. Ectopic *Snail1* expression can be observed in the mutant embryos leading to an extended region of EMT and cell ingression. The dotted lines in the whole mounted embryos indicate the level of the sections shown in the lower panels. Red brackets indicate the areas of cell delamination in the enlarged pictures and in the diagrams, which schematically show the defective phenotype in the gastrulating embryos. Dotted lines in the diagram indicate the midline.

Next, we wondered whether our observations in the chick could be extended to other systems, and we compared the expression of *Sox3* and *Snail* genes in the mouse gastrula. Snail2 is not expressed in the primitive streak or early mesoderm in mouse embryos because of a reshuffling of *Snail1* and *Snail2* expression domains during evolution (Sefton et al., 1998; Locascio et al., 2002). Thus, we compared the expression patterns of *Snail1* and *Sox3*. As previously described, *Sox3* is strongly expressed in the epiblast (Wood and Episkopou, 1999; Figure 7A), and *Snail1* is expressed at the primitive streak and in the mesodermal cells delaminating from it, but not in the epiblast (Sefton et al., 1998; Figure 7A). This complementary expression is compatible with the idea that, in the mouse, Snail1 and Sox3 repress each other's transcription. This is also reinforced by the conservation of the Snail-binding site in the mouse *Sox3* promoter and the presence of two consensus Sox DNA-binding sequences located in conserved regions of the mouse *Snail1* promoter (Figure S1).

To directly examine the influence of Sox3 on *Snail1* expression, we used wild-type cells (CCE cells; Robertson et al., 1986; Keller et al., 1993) or Sox3 null mouse ES cells (M. Parsons, C.W., and R.L.-B., unpublished data) that were analyzed after 5 or 8 days in the presence or in the absence of LIF, the latter allowing the generation of embryoid bodies. *Snail1* expression is activated in Sox3-deficient embryoid bodies (Figure 7B), suggesting that the absence of Sox3 leads to a derepression of *Snail1* expression. Concomitant with *Snail1* activation, *E-cadherin* was downregulated (Figure 7B), indicating the presence of aberrant expression of Snail1 and downregulation of E-cadherin, as confirmed by immunohistochemistry (Figure 7C). Furthermore, although embryoid bodies derived from wild-type ES cells look round and show a compact morphology, those derived from *Sox3* mutant ES cells are irregular. We excluded both the possibility of these cells dying and the existence of differences in the rate of cell proliferation (Figure S2). Rather, they are healthy cells that appear to disaggregate, resembling a process of EMT (Figure 7C). These results pointed to a conserved antagonistic relationship between Sox3 and Snail1 in the mouse.

Further support for the conservation comes from the observation of gastrulation defects in chimeras obtained after injection of *Sox3* null ES cells in mouse blastocysts (M. Parsons, C.W., and R.L.-B., unpublished data). *Sox3* is located on the X chromosome; therefore, targeted XY ES cells are null for *Sox3*. However, this gastrulation defect was observed in the context of chimeric embryos, because ubiquitous deletion of a floxed allele of *Sox3* by βactinCre results in generation of live animals (Rizzoti et al., 2004). This suggests that the presence of *Sox3* null cells in a wild-type host embryo is incompatible with a compensation mechanism that allows normal gastrulation in an entirely *Sox3* null embryo. The latter is likely to involve Sox2, which, in contrast to the situation in the chick, shows similar expression at these stages in the mouse (Avilion et al., 2003). Therefore, if the antagonistic relationship between Sox3 and Snail is conserved in the mouse as the EB experiments indicate, then gastrulation defects similar to those we described here in the chick should be observed after lowering the *Sox2* dose in the *Sox3* mutant. Because *Sox2* null embryos die at peri-implantation stages, we analyzed *Sox3* null; *Sox2* heterozygous embryos and indeed, we found ectopic *Snail1* expression and deformed embryos with an extended area of cell delamination at the primitive streak (Figure 7D; n = 3). These results support and extend those found in the *Sox3* null EBs, indicative that the antagonistic relationship with Snail is conserved in the mouse.

### Conservation of the Sox3/Snail Antagonistic Relationship in Human Cancer Cells

**Figure 8:**
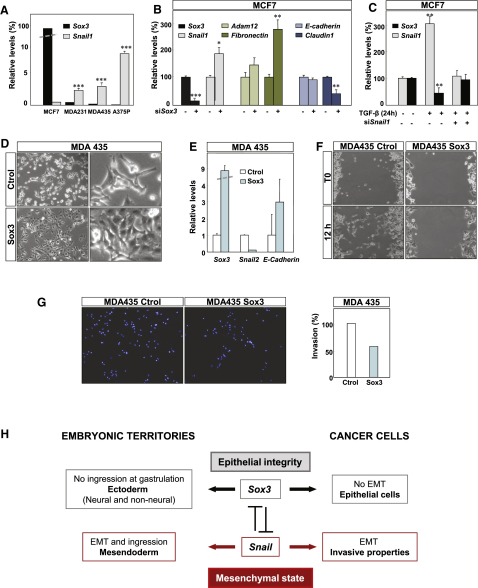
The Antagonistic Relationship between Sox3 and Snail Factors Is Conserved in Human Cancer Cells (A) Expression of *Sox3* and *Snail1* in different human cancer cell lines. Epithelial MCF7 cells express high levels of *Sox3* and low levels of *Snail1*. Conversely, mesenchymal MDA231, MDA435, and A375P lines are almost devoid of *Sox3* transcripts and show high *Snail1* expression. (B) Efficient *Sox3* downregulation by specific siRNA in MCF7 cells is accompanied by an increase in *Snail1* and associated mesenchymal markers *Adam12* and *Fibronectin* and a decrease in the epithelial marker *Claudin1*. (C) TGF-β mediated *Snail1* induction in MCF7 cells is accompanied by a decrease in *Sox3* expression. This decrease is Snail1-dependent, as it is prevented by transfection with *Snail1* siRNA. (D and E) Sox3 stable overexpression in MDA435 leads to morphological changes compatible with a partial mesenchymal to epithelial transition (D). These morphological changes are associated with a decrease in *Snail2* expression and an increase in *E-cadherin* expression (E). (F) The migratory behavior of MDA435-Sox3 stable transfectants was tested in culture using a wound healing assay. Control MDA435 cells cover the wound in 12 hr, in clear contrast with Sox3-expressing cells. (G) Invasive behavior of MDA435-Sox3 cells. The nuclei of cells that invaded the collagen matrix were stained with DAPI and quantified. MDA435 cells expressing Sox3 significantly decreased their invasive properties. (H) Diagram showing the relationship between Snail and Sox3 factors in gastrulating embryos and in cancer cells. In gastrulating embryos, the mutual repression between Sox3 and Snail1 regulates the decision to ingress at the primitive streak. Sox3 expressing cells do not ingress, ensuring the development of ectodermal derivatives in developing embryos. In cancer cells, Snail induces EMT and its expression correlates with invasive properties. Sox3 represses Snail, thereby preventing EMT to maintain epithelial integrity.

Because Snail factors are also implicated in the repression of the epithelial phenotype and the induction of EMT during tumor progression (Thiery et al., 2009), we checked whether human cancer cell lines also show an antagonistic relationship between Snail and Sox3 transcription factors. We examined one epithelial cell line derived from breast carcinoma (MCF7) and three independent mesenchymal and invasive lines, two of them also derived from breast tumors (MDA231 and MDA435) and one from a melanoma (A375P). *Sox3* is strongly expressed in MCF7 cells and it is absent from A375P, MDA231, and MDA435. Conversely, *Snail1* expression is high in MDA231, MDA435, and A375P and very low in MCF7 cells (Figure 8A). To address whether the expression of *Snail1* and *Sox3* is interdependent, we first interfered with *Sox3* expression in MCF7 cells by transfecting specific siRNAs (see Experimental Procedures). We found an efficient *Sox3* downregulation concomitant with an increase in *Snail1* expression (Figure 8B) accompanied by the decrease in the epithelial marker *Claudin1*, and an increase in the mesenchymal markers *Adam12* and *Fibronectin* (Figure 8B). We did not observe a reduction in E-cadherin levels in this transient downregulation of Sox3 expression. Importantly, because MCF7 cells do not express significant levels of *Snail1*, we added 2 ng/ml of its potent inducer TGFβ to the cultures. This leads to three-fold induction of *Snail1* expression, which correlates with a 50% decrease of *Sox3* transcripts (Figure 8C). Interestingly, the changes in *Sox3* expression were dependent on Snail1, because *Sox3* transcript levels remained unaffected in the presence of a *Snail1*-specific siRNA, which prevented Snail1 induction by TGFβ (Figure 8C). To examine whether the interdependent changes in gene expression had an impact on cell morphology and behavior, we infected the mesenchymal MDA435 cells with a retrovirus containing the Sox3 coding sequence to generate MDA435-Sox3 cells stably expressing this transcription factor. We observed morphological changes compatible with a partial mesenchymal-to-epithelial transition (Figure 8D), concomitant with the decrease in *Snail2* expression and the reactivation of *E-cadherin* transcription (Figure 8E). We next examined cell behavior and found that Sox3 induced a dramatic decrease in cell motility as assessed in a wound healing assay in culture (Figure 8F) and a decrease cell invasion in collagen gels (Figure 8G). In summary, the antagonistic relationship between Snail1 and Sox3 is maintained in cancer cells, and their relative expression correlates with their morphological, motility and invasive properties.

## Discussion

One of the earliest cellular decisions in metazoans is the subdivision of the early embryo into the domains that will give rise to the different embryonic layers. In amniote embryos, the first subdivision occurs at the primitive streak, where ingressing cells will later become mesoderm and endoderm and the noningressing cells will become ectoderm. Here we show that the partitioning of these cellular domains at early primitive streak stages is regulated by interactions between two transcription factors, Snail and Sox3, which direct ingressing versus noningressing behaviors, respectively. In the chick embryo, Snail2 and Sox3 act as mutual transcriptional repressors; cells that express high levels of Snail2 are devoid of *Sox3* transcripts and ingress through the primitive streak. In contrast, cells expressing high levels of Sox3 lack Snail2 expression and stay in the epiblast. Both Sox3 ectopic expression and Snail2 inhibition close to the primitive streak prevent cell ingression while still permitting the movement toward the midline. In turn, Snail2 overexpression or Sox3 inhibition increases cell ingression at the primitive streak. This indicates that the interplay between Snail2 and Sox3 controls the delamination from the epiblast, thereby ensuring the subdivision of the embryo into two populations, ingressing and noningressing, that will later give rise to the mesendoderm and to much of the ectoderm, respectively. At later stages, the posterior neural tube arises from a population of bipotent axial stem cells set aside within the region of the tail bud (Wilson et al., 2009). These stem cells can become posterior neural tissue or undergo EMT to become paraxial mesoderm, and their ingression depends on the repression of Sox2 by Tbx6, as recently shown in the mouse (Takemoto et al., 2011).

Our data show that Sox3 and Snail behave as direct repressors of each other. Snail is a well-known transcriptional repressor that controls cell movements both in embryonic development and tumor progression (Thiery et al., 2009). Interestingly, although the genes of the SoxB1 subgroup (Sox1, Sox2, and Sox3) are usually described as transcriptional activators (Uchikawa et al., 1999), here we show that like the SoxB2 subgroup genes (Sox14 and Sox21), Sox3 can also function as a transcriptional repressor depending on the context, as also described for Sox2 during the differentiation of ES cells (Navarro et al., 2008). We demonstrate that Sox3 directly binds to the promoter of its target gene, *Snail2*. This is in agreement with recent findings in the zebrafish embryo showing that Sox3 can repress the expression of *Bozozok*, a homeobox gene important for the formation of the dorsal organizer and subsequent gastrulation movements (Shih et al., 2010). We also show that Sox3 represses the activity of a *Snail2* promoter construct in vivo, now confirming that, in addition to a transcriptional activator, Sox3 can be considered as a bona fide transcriptional repressor, depending on context.

Our description of the involvement of Sox3/Snail interactions in defining embryonic territories in the chick is consistent with the gastrulation defects observed in chimeras obtained after injection of Sox3 null ES cells in mouse blastocysts. This gastrulation phenotype observed in the chimeras was difficult to explain, considering that Sox3 is never expressed in the mesoderm at gastrulation stages. However, our data on the regulation of *Snail1* and *Sox3* expression in embryoid bodies obtained from mouse *Sox3* null ES cells and our analysis of gastrulating *Sox3* null; *Sox2* heterozygous embryos provide a simple explanation for the gastrulation defects first observed in the chimeras containing Sox3 null ES cells and confirm the conservation of the interplay between Sox3 and Snail in defining ectodermal and mesendodermal territories.

Our data further clarify recent data showing multiple defects, including gastrulation defects, in zebrafish embryos after downregulation of the full complement of SoxB1 genes (Okuda et al., 2010). The downregulation of individual SoxB1 genes does not give rise to severe defects in the fish, reflecting the overlap in the expression patterns of several family members, particularly for *Sox3* and the fish-specific *Sox19a* and *19b* (Okuda et al., 2006). In addition, in the fish embryo, the formation and migration of mesendoderm does not involve a full EMT, but rather cells very quickly re-express the homolog of E-cadherin (Cdh1) and migrate as a cohesive group (Montero et al., 2005). In the fish, Cdh1 re-expression is necessary for the proper migration of the mesendodermal cells (Montero et al., 2005) and indeed it occurs concomitantly with the loss of snail1a, which is only transiently expressed in the involuting mesoderm (Blanco et al., 2007). Furthermore, a dominant-negative form of Sox3 in the zebrafish induces the formation of multiple organizers (Shih et al., 2010), indicating that in the fish, as we show here in the amniote embryo, Sox3 needs to be downregulated for gastrulation to proceed normally. Interestingly, both in fish and *Xenopus*, gastrulation starts concomitantly with the transient activation of *Snail* expression (Blanco et al., 2007; Mayor et al., 2000). Thus, the antagonism between Sox3 and Snail factors shown here in the amniote embryo, although not directly examined in zebrafish or *Xenopus*, may contribute to the initiation of the gastrulation process and may therefore be conserved not only in amniotes but also in anamniote embryos.

A crucial issue that also emerges at gastrulation is the necessary coordination between cell fate and cell behavior. Snail has been considered as a mesodermal determinant from studies in *Drosophila* where it is a repressor of nonmesodermal genes. However, we would like to argue that the role of Snail is independent from cell fate determination, its main role being the regulation of cell behavior. Our data indicate that Snail2 can trigger an ectopic EMT and cell delamination from the epiblast but is unable to induce the expression of mesodermal or endodermal markers. This is in agreement with data from *Drosophila* indicating that low levels of snail do not repress nonmesodermal genes in the presumptive mesoderm while still able to promote invagination (Ip et al., 1994) and it is also consistent with data from the mouse showing that Snail1-deficient embryos can form mesodermal tissue that expresses Brachyury and Tbx6 but these cells are unable to migrate due to defects in EMT and the continued expression of E-cadherin (Carver et al., 2001; Murray and Gridley, 2006). Indeed, fate determination and cell delamination seem to be two independent processes, both driven by FGF signaling through FGFR1 in the gastrulating mouse, exerted on one hand by maintaining the expression of *Snail* and on the other by controlling the expression of the mesodermal genes *Brachyury* and *Tbx6* (Ciruna and Rossant, 2001). Thus, our data show that the definition of the two main embryonic territories in the early gastrulating embryo, namely the ectoderm and the mesendoderm, is governed by the control of cell behavior driven by the antagonistic role between Snail and Sox3 factors independently from cell specification, which is concomitantly coordinated by FGF signaling. Recent data from ascidian embryos indicate that the subsequent decision for the mesendoderm to subdivide into mesoderm or endoderm is determined by the asymmetric partitioning of the Not transcription factor in the cells destined to become mesodermal, a mechanism that is likely to be conserved in vertebrates because Not has been already described in *Xenopus*, fish, and chick embryos (Takatori et al., 2010).

Pioneer work in the chick embryo showed that when a subset of ectodermal cells is specified to become the nervous system, the expression of another transcription factor, Churchill, acts as an important switch, preventing the ingression of prospective neural plate cells through the anterior part of the primitive streak from late stage HH4 onward (Sheng et al., 2003). The Sox3/Snail switch that we describe here to control cell ingression occurs before neural induction and before the onset of Churchill expression. The two mechanisms are sequentially implemented in the embryo, with the Snail/Sox3 axis acting first to ensure that a subpopulation of ectodermal cells stays in the epiblast. Therefore, the interplay between Sox3 and Snail controls the first subdivision of embryonic territories. Subsequently, upon neural induction, Churchill ensures that the subpopulation of ectodermal cells already specified as neural precursors do not ingress through the primitive streak to become mesoderm or endoderm.

Finally, our data obtained in human cancer cell lines suggest that the mutual repression between Sox3 and Snail is also in place. Epithelial tumor cells express high levels of *Sox3* and low levels of *Snail1*, and the opposite is true for mesenchymal tumor cells. Not only are *Snail1* and *Sox3* expression levels associated with the morphological and invasive phenotype, but also interference with Sox3 or Snail1 expression induces reciprocal changes in their expression, compatible with the existence of a loop of mutual repression, as described in embryos. These data may have important implications in tumor biology, as Snail reactivation and EMT contributes to the first steps of the metastatic cascade in carcinomas and it is considered a target of anti-invasive drugs (Thiery et al., 2009; Nieto, 2011). Therefore, it is important to identify not only how Snail is reactivated in tumors but also to identify its negative regulators. We propose Sox3 as a likely candidate.

## Experimental Procedures

### Chick Embryos and Explant Cultures

Fertilized hen eggs were purchased from Granja Gilbert (Tarragona, Spain). The eggs were incubated and opened, and the embryos were explanted for EC culture as described elsewhere (Flamme, 1987; Chapman et al., 2001). Embryos were staged according to Eyal-Giladi and Kochav (1976) (EG) and Hamburger and Hamilton (1951) (HH), selecting HH2 embryos for experiments.

### Mouse Embryos, ES Cells, and Human Cell Lines

Mouse embryos were obtained by crossing C57 and CBA mice. Embryos dissected at 7.5 dpc were fixed overnight in 4% paraformaldehyde. CCE mouse ES cells (Robertson et al., 1986; Keller et al., 1993) were cultured in cell culture dishes with DMEM (Invitrogen) supplemented with 10% serum and LIF (1000 U/ml, Chemicon International). Embryoid bodies were grown on bacteriological grade plastic dishes in the same medium in the absence of LIF, and total RNA was extracted with Trizol (Life Technologies) after different times in culture. MCF7, MDA231, MDA435, and A375P human tumor cell lines were purchased from the ATCC (Virginia, USA) and were cultured in DMEM supplemented with 10% heat inactivated serum and 0.1% penicillin-streptomycine (Invitrogen). Cells were transfected with negative control for siRNAs or with those directed against *Snail1* (3 sequences tested) or *Sox3* (5 sequences tested) using Lipofectamine RNAiMAX following the manufacturer's instructions (Invitrogen). For RNA/Lipofectamine complex formation, siRNAs were used at a working concentration of 100 nM. Because they were able to downregulate expression with different efficiencies, we present the data obtained with the most efficient oligonucleotide in each case, whose sequences are shown in Table S1. RNA was isolated 2 days after transfection for the Sox3 interference experiment. When indicated, 2 ng/ml TGFβ was added to the cells 24 hr after Snail1 siRNA transfection. Total RNA was obtained at 1 hr, 24 hr, and 48 hr after TGFβ administration, and in all cases *Snail1* induction was impaired (Figure S3). Total RNA was purified using the illustra RNAspin Mini kit including DNaseI treatment (GE Healthcare). Stealth siRNA (Invitrogen) sequences were as follows: 5′-UCCCAGAUGAGCAUUGGCAGCGAGG-3′ against human *Snail1* (*SNAI1*) and 5′-AGUUCCAGGGUUAUUCUGUUACAUU-3′ against human *SOX3.*

### Viral Production and Generation of Sox3 Stable Expressing Cells

Retroviral production and infection were carried out as previously described (Mani et al., 2008). After infection, MDA435 cells expressing either pBabe-PURO or pBabe-PURO-Sox3 were selected with 10 μg/ml puromycin for 2 weeks.

### Migration and Invasion Assays

For migration assays, cells were seeded in six-well culture dishes at a density of 1 × 10^6^ cells/well. A wound was made in the center of the culture 24 hr later, and phase-contrast pictures were taken at different time intervals. Invasion assays on collagen type-IV gels were performed as previously described (Cano et al., 2000). Briefly, 6 × 10^4^ cells of each type were seeded onto the upper surface of the filters. After 12 hr of incubation, cells attached in the lower part of the filters were fixed in methanol, stained with 4,6-diamidinophenylindole (DAPI) and counted.

### Electroporation of Chicken Embryos

Explanted embryos at HH2 were placed, vitelline membrane and filter paper down, over an electroporation chamber (NEPAGEN) containing a platinum electrode connected to the negative pole. A solution containing expression plasmids (2 mg/ml in PBS with 0.1% Fastgreen and 6% sucrose) was injected between the vitelline membrane and the epiblast. An anodal electrode was placed over the hypoblast to cover the injected area. A train of electric pulses (5 pulses, 4 Volts, 50 ms, and 0.5 Hz) was applied using an Intracept TSS10 pulse stimulator (Intracell). In all experiments, the nonelectroporated right side of the embryo was used as a control. The embryos were then cultured at 38°C (Chapman et al., 2001) to the desired stage. Embryos were photographed with a Leica MZFLIII dissecting microscope to record GFP expression and fixed overnight in 4% paraformaldehyde (PFA) in PBS at 4°C to be processed for in situ hybridization or immunohistochemistry. Cell death that might have resulted from the electroporation procedure was excluded as a factor to influence cell behavior (Figure S2).

### Time-Lapse Confocal Imaging

Six hours after electroporation, chicken embryos at stage HH3+ were washed in PBS and placed into a glass-bottom culture 35 mm Petri dish (MatTek) containing egg albumin. The dish was then located into an incubation chamber at 38°C surrounding a Leica inverted confocal microscope for image acquisition. One image was captured each 10 min for a total of 8 hr. Movies were assembled using the ImageJ software (http://rsbweb.nih.gov/ij/). Individual cells were tracked using the “Manual Tracking” plug-in by F. Cordelières (http://rsbweb.nih.gov/ij/plugins/track/track.html). Ingression was quantified as the percentage of ingressing and noningressing cells after tracking 20 cells per field in three fields per movie.

### DNA Constructs

pCX-Snail2, pCX-DN-Snail2, and pCX-GFP expression vectors were previously described (Morales et al., 2007). Full-length Sox3 or a truncated dominant negative form of Sox3 similar to that previously described for the *Sox2 Xenopus* gene (Kishi et al., 2000) were cloned in pCX at the EcoRI restriction site. *Snail2* and *Sox3* promoters were PCR amplified from chick genomic DNA using Phusion High Fidelity DNA polymerase (Finnzyme) (see Table S1 for primers sequences), sequenced, and inserted in pGL2 basic using the KpnI and MluI restriction sites. For viral production, the coding sequence of human Sox3 was amplified by PCR (see Table S1 for primers sequences) and inserted in the pBabe-PURO vector using the EcoRI restriction site.

### Whole-Mount In Situ Hybridization

Whole-mount in situ hybridization was carried out as described previously (Nieto et al., 1996) omitting the proteinase K treatment. Digoxigenin-labeled probes were synthesized from the full-length chicken cDNAs of *Brachyury*, *Otx2*, *RhoB* (Liu and Jessell, 1998), and *Snail2* (Nieto et al., 1994) and from Expressed Sequence Tags (EST; Boardman et al., 2002) for Dlx5 (ChEST808h7), Tbx6L (ChEST90h8), and Sox17 (pgr1n.pk001.g24; Chapman et al., 2007). Chicken and mouse Sox3 sequences were PCR amplified from chicken and mouse genomic DNA (Table S1) and cloned in pGEMT-easy. Mouse Snail1 probe was previously described (Sefton et al., 1998). Hybridized probes were detected using an alkaline phosphatase-conjugated anti-digoxigenin antibody (Roche, 1:1000) in the presence of NBT/BCIP substrates (Roche). For whole-mount fluorescent in situ hybridization, embryos were processed as previously described (Acloque et al., 2008). Briefly, probes were labeled using digoxigenin- or fluorescein-coupled nucleotides (Roche, 1:1000) and were sequentially developed with POD-conjugated anti-fluorescein or anti-digoxigenin antibodies (Roche). Peroxidase activity was successively detected with the TSA-plus Cy3 and Fluorescein kits (Perkin Elmer). In some cases, the embryos were subjected to immunostaining with anti-GFP antibody (Invitrogen, 1:1000). After hybridization and/or immunohistochemistry, embryos were fixed in 4% paraformaldehyde in PBS, washed, and photographed under a Leica M10 dissecting scope. Some embryos were subsequently embedded in paraffin (Fibrowax) or gelatin, sectioned at 10 μm or 40 μm, respectively, and photographed using a Leica DMR microscope under Nomarski optics and equipped with an Olympus DP70 digital camera.

### Immunohistochemistry

For immunohistochemistry, electroporated embryos were fixed in PFA 4% in PBS. For laminin detection, 10 μm cryostat sections were treated with 0.1% Triton X-100 (Sigma) in PBS, blocked with 10% FBS in PBS and incubated overnight at 4°C with anti-laminin (primary antibody (Sigma) at 1:1000 dilution. For E-cadherin detection, chick embryos were embedded in paraffin and sectioned at 8 μm. Immunostaining was performed by standard procedures using anti-GFP antibody (rabbit polyclonal, Invitrogen; 1:500) and anti-E-cadherin (mouse monoclonal, BD Bioscience; 1:250). After washing, sections were incubated for 1 hr with Alexa488 (Invitrogen, 1:1000) and Cy3 conjugated (Jackson; 1:1000) secondary antibodies and photographed using a Leica DMR microscope.

Embryoid bodies were fixed with ice-cold methanol, rehydrated, and immunostained by standard procedures using anti-E-cadherin (mouse monoclonal ECCD-2, Takara; 1:250) or anti-Snail1 (Abcam). Images were acquired using a Leica inverted confocal microscope.

### Chromatin Immunoprecipitation

Chicken embryos were electroporated either with GFP and control myc-Tag, GFP and myc-Snail2, or GFP and myc-Sox3 expression plasmids. Eight hours after electroporation, GFP-positive tissues were dissected from HH5 embryos. Tissues were crosslinked with 1% formaldehyde in PBS for 10 min at room temperature and quenched with 0.125 M glycine for 5 min at room temperature. Tissues were then washed three times in PBS and resuspended in SDS lysis buffer (1% SDS, 10 mM EDTA, and 50 mM Tris [pH 8.1]) using 100 μl of buffer for a pool of 15 embryos (corresponding to approximately 1 × 10^5^ cells). Lysates were sonicated in an ultrasonic cell disrupter (Bioruptor, Diagenode SA, Belgium) for 8 min, with alternating 30 s off and on, frozen in liquid nitrogen and stored at −80°C. Each sonicated lysate (about 1 × 10^5^ cells) was diluted in 900 μl of buffer (0.01% SDS, 1.1% Triton X-100, 1.2 mM EDTA, 16.7 mM Tris [pH 8.1], and 167 mM NaCl) in the presence of protease inhibitors. Ten microliters was recovered as the input fraction, and the rest was divided and incubated overnight at 4°C with anti-myc ChIP grade (ab9132, Abcam, UK), anti-H3 ChIP grade (ab1791, Abcam, UK), or rabbit IgG control (Diagenode, Belgium) using 1 μg of antibody for tissue lysate. Immunoprecipitation of crosslinked Protein/DNA was performed adding 60 μl of a slurry of Protein A Agarose beads (Roche) previously saturated with salmon sperm DNA (1 mg/ml) and BSA (1 mg/ml). Complexes were washed using Low Salt Washing Solution (0.1% SDS, 1% Triton X-100, 2 mM EDTA, 20 mM Tris [pH 8.1], and 150 mM NaCl), High Salt Washing Solution (0.1% SDS, 1% Triton X-100, 2 mM EDTA, 20 mM Tris [pH 8.1], and 500 mM NaCl), LiCl Washing Solution (0.25 M LiCl, 1% IGEPAL, 1% deoxycholic acid, 1 mM EDTA, and 10 mM Tris [pH 8.1]), and two times TE Solution (10 mM Tris [pH 8.1] and 1 mM EDTA). Protein/DNA complexes were then eluted in 200 μl of elution buffer (1% SDS and 100 mM NaHCO_3_) and dissociated by incubating the samples at 65°C for 5 hr in the presence of 200 mM NaCl (added to the elution buffer). DNA was then purified using affinity columns and amplified by PCR and real-time PCR using H3 samples as a reference.

### PCR and Real-Time PCR

DNA obtained from the ChIP experiments was amplified using QPCR PromSox3 SnailRE and QPCR PromSnail2 SoxRE primers (see Table S1 for sequences). Reverse transcription was performed using random priming and Superscript Reverse Transcriptase (Life Technologies), according to the manufacturer's guidelines. Real-time PCRs were performed using Absolute SYBR Green mix (Thermo Scientific) in a Step One Plus machine or ABI PRISM 7500 thermocycler (Applied Biosystems). MCF7, MDA231, and MDA435 breast cancer and A375P melanoma cell lines cDNAs were amplified to examine human *Sox3* and *Snail1* expression, using 36B4 as internal control (Côme et al., 2006) and applying relative quantification using the 2-ΔΔCt method. For embryoid bodies variations in input RNA were corrected by subtracting the number of PCR cycles obtained for β-actin. All primers sequences are described in Table S1.

### Luciferase Assays

Chicken embryos were electroporated with pRL-CMV (Promega) as an internal control and either pGL2b (Promega), pGL2-promSox3, pGL2 delpromSox3, pGL2-promSnail2, or pGL2 delpromSnail2, in the presence or absence of pCX-Snail2 (for Sox3 promoter experiments) or pCX-Sox3 (for Snail2 promoter experiments). For each assay, three electroporated embryos were pooled to get one measurement and experiments were made in triplicate (9 embryos per experiment). Tissues were lysed using Passive lysis buffer (Promega) and activity measured with Dual luciferase assays (Promega) using a Berthold luminometer.

### Statistical Analysis

In figures including statistical analyses, the values represent mean + SD of three independent experiments (ANOVA analysis, ^∗^ p < 0.1, ^∗∗^ p < 0.01, ^∗∗∗^ p < 0.001).

## Supplemental Information

Document S1. Three Figures and One Table

Movie S1. Cell Ingression at the Primitive Streak in the Chick Gastrula Stage 2 (H&H) chicken embryos were electroporated to overexpress GFP on the left side of the epiblast. Time-lapse movies were recorded 6 hr after electroporation for 8 hr, taking one picture each 10 min. The movies were recorded from stage 3+ to stage 5 (H&H) and show the ingression of GFP-positive cells at the primitive streak (PS). Note the typical movement of cells converging to the PS, ingressing and migrating away from the streak.

Movie S2. Tracking of Individual Cells Ingressing at the Primitive Streak in the Chick Gastrula Some individual cells from Movie S1 were tracked using the “Manual Tracking” plug-in by F. Cordelières (http://rsb.info.nih.gov/ij/plugins/track/track.html). Tracks show the typical movement of cells converging to the PS, ingressing and migrating away from the streak.

Movie S3. Cells Overexpressing Snail2 Ingress Massively at the Primitive Streak in the Chick Gastrula Stage 2 (H&H) chicken embryos were electroporated to overexpress GFP and Snail2 on the left side of the epiblast. Time-lapse movies were recorded 6 hr after electroporation at HH3+ for 8 hr, taking one picture each 10 min. Snail2/GFP-positive cells converge to the PS and ingress massively.

Movie S4. Tracking of Individual Cells Overexpressing Snail2 Some individual cells from Movie S3 were tracked using the “Manual Tracking” plug-in by F. Cordelières (http://rsb.info.nih.gov/ij/plugins/track/track.html). Cell tracks show the convergence of cells to the PS and their ingression at the PS.

Movie S5. Cells Overexpressing Sox3 Do Not Ingress at the Primitive Streak in the Chick Gastrula Stage 2 (H&H) chicken embryos were electroporated to overexpress GFP and Sox3 on the left side of the epiblast. Time-lapse movies were recorded 6 hr after electroporation at HH3+ for 8 hr, taking one picture each 10 min. Sox3/GFP-positive cells converge to the PS but fail to ingress.

Movie S6. Tracking of Individual Cells Overexpressing Sox3 Shows that They Do Not Ingress at the Primitive Streak in the Chick Gastrula Some individual cells from Movie S3 were tracked using the “Manual Tracking” plug-in by F. Cordelières (http://rsb.info.nih.gov/ij/plugins/track/track.html). Cell tracks show the convergence of cells to the PS and their failure to ingress.

Movie S7. Cells Overexpressing a Dominant-Negative Form of Snail2 Do Not Ingress at the Primitive Streak in the Chick Gastrula Stage 2 (H&H) chicken embryos were electroporated to overexpress GFP and a dominant-negative form of Snail2 (DN-Snail2) on the left side of the epiblast. Time-lapse movies were recorded 6 hr after electroporation at HH3+ for 8 hr, taking one picture each 10 min. Similarly to cells overexpressing *Sox3*, DN-Snail2/GFP-positive cells converge to the PS but fail to ingress.

Movie S8. Tracking of Individual Cells Overexpressing a Dominant-Negative Form of Snail2 Shows that They Do Not Ingress at the Primitive Streak in the Chick Gastrula Some individual cells from Movie S5 were tracked using the “Manual Tracking” plug-in by F. Cordelières (http://rsb.info.nih.gov/ij/plugins/track/track.html). Cell tracks show the convergence of cells to the streak and their failure to ingress.

Movie S9. Cells Overexpressing a Dominant-Negative Form of Sox3 Ingress Massively at the Primitive Streak in the Chick Gastrula Stage 2 (H&H) chicken embryos were electroporated to overexpress GFP and a dominant-negative form of Sox3 (DN-Sox3) on the left side of the epiblast. Time-lapse movies were recorded 6 hr after electroporation at HH3+ for 8 hr, taking one picture each 10 min. Similarly to cells overexpressing Snail2, DN-Sox3/GFP-positive cells converge to the PS and ingress massively.

Movie S10. Tracking of Individual Cells Overexpressing a Dominant-Negative Form of Sox3 Some individual cells from Movie S9 were tracked using the “Manual Tracking” plug-in by F. Cordelières (http://rsb.info.nih.gov/ij/plugins/track/track.html). Cell tracks show the convergence of cells to the streak and their ingression at the PS.

Movie S11. Cell Movements after Electroporation of Both Sox3 and Snail2 Are Reminiscent of Those Observed in Control Embryos Chicken embryos were electroporated on the left side of the epiblast at stage HH2. Time-lapse movies were recorded 6 hr after electroporation for 8 hr, taking one picture each 10 min. The movies were recorded from stage 3+ to stage 5 (H&H) and show the ingression of GFP-positive cells at the primitive streak.

Movie S12. Tracking of Individual Cells in Embryos Electroporated with Both Sox3 an,d Snail2 Shows that They Do Ingress at the Primitive Streak in the Chick Gastrula Some individual cells from Movie S11 were tracked using the “Manual Tracking” plug-in by F. Cordelières (http://rsb.info.nih.gov/ij/plugins/track/track.html). Cell tracks show the cells converging to the PS, ingressing and migrating away from the streak.
