# Supplementary material for: Reciprocal Repression between Sox3 and Snail Transcription Factors Defines Embryonic Territories at Gastrulation
Source: Dev Cell. 2011 Sep 13;21(3):546–58. doi: 10.1016/j.devcel.2011.07.005 (PMC3256632; doi:10.1016/j.devcel.2011.07.005)
Supplement: Document S1. Three Figures and One Table [file mmc1.pdf]

Developmental Cell, Volume *21*

Supplemental Information

Reciprocal Repression between Sox3 and Snail Transcription Factors Defines Embryonic Territories at Gastrulation

Hervé Acloque, Oscar H. Ocaña, Ander Matheu, Karine Rizzoti, Clare Wise, Robin Lovell-Badge, and M. Angela Nieto

Inventory of Supplemental Information

Our manuscript includes

1.- Supl. Fig. 1 is directly associated with Figure 5

2.- Supl. Fig. 2 is directly associated with Figure 7C

3.- Supl. Fig. 3 is directly associated with Figure 8C

4.- Supplemental Table I contains the sequences of oligonucleotides used throughout the work.

5.- Twelve Supplemental movies. The movies are directly associated with panels in Fig. 4.

6.- There is one text document containing the legends to the movies.

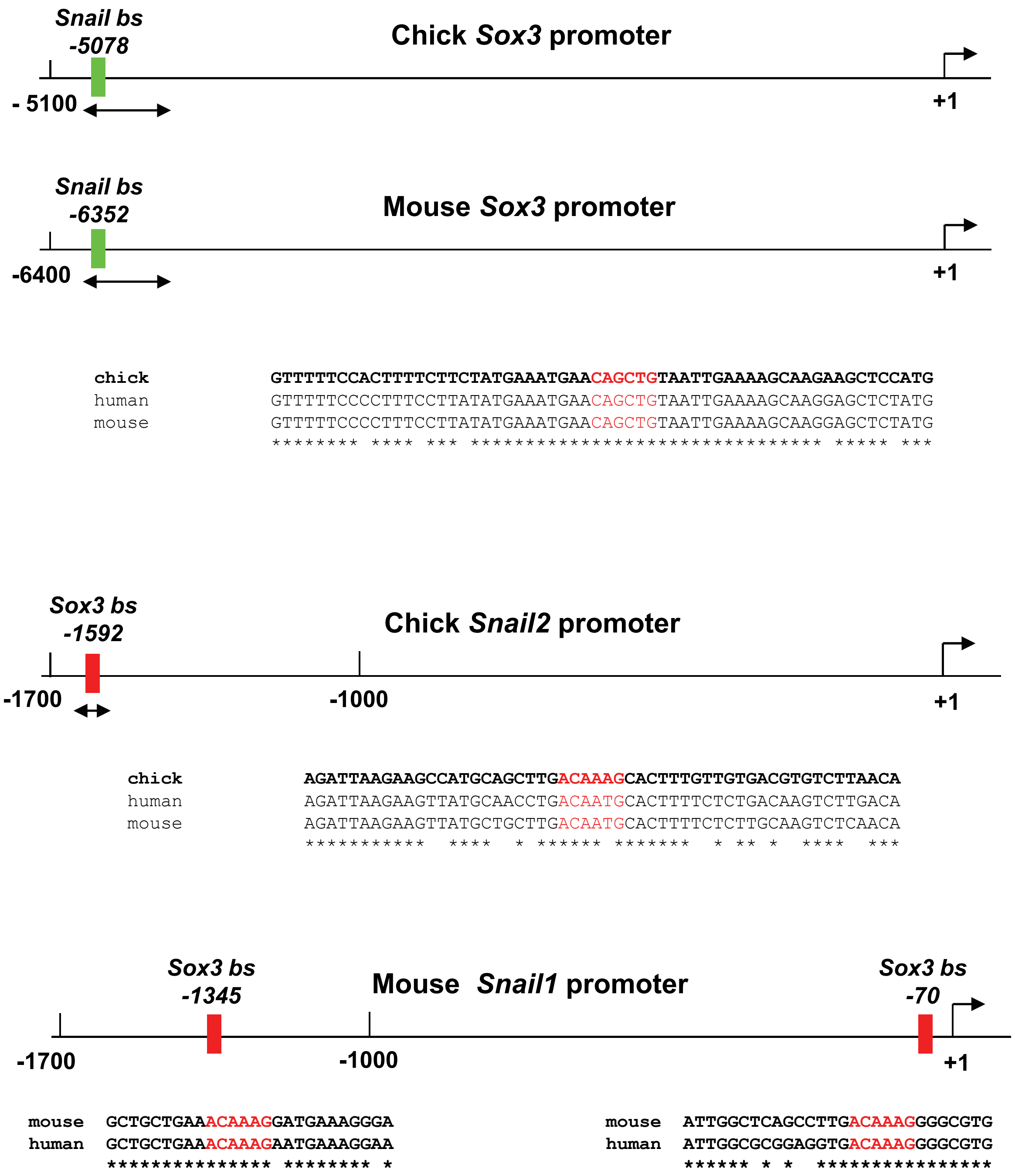

Supplementary Figure 1

Schematic representation of *Sox3*, *Snail1* and *Snail2* promoters. Double arrows highlight conserved regions between chicken, mouse and human genes and green and red boxes map conserved response elements for Snail (CASSTG) and SoxB (WCAAWG), respectively. Alignments of sequences including the boxes are shown below. Positions relative to the Transcription Start Site are indicated above the boxes.

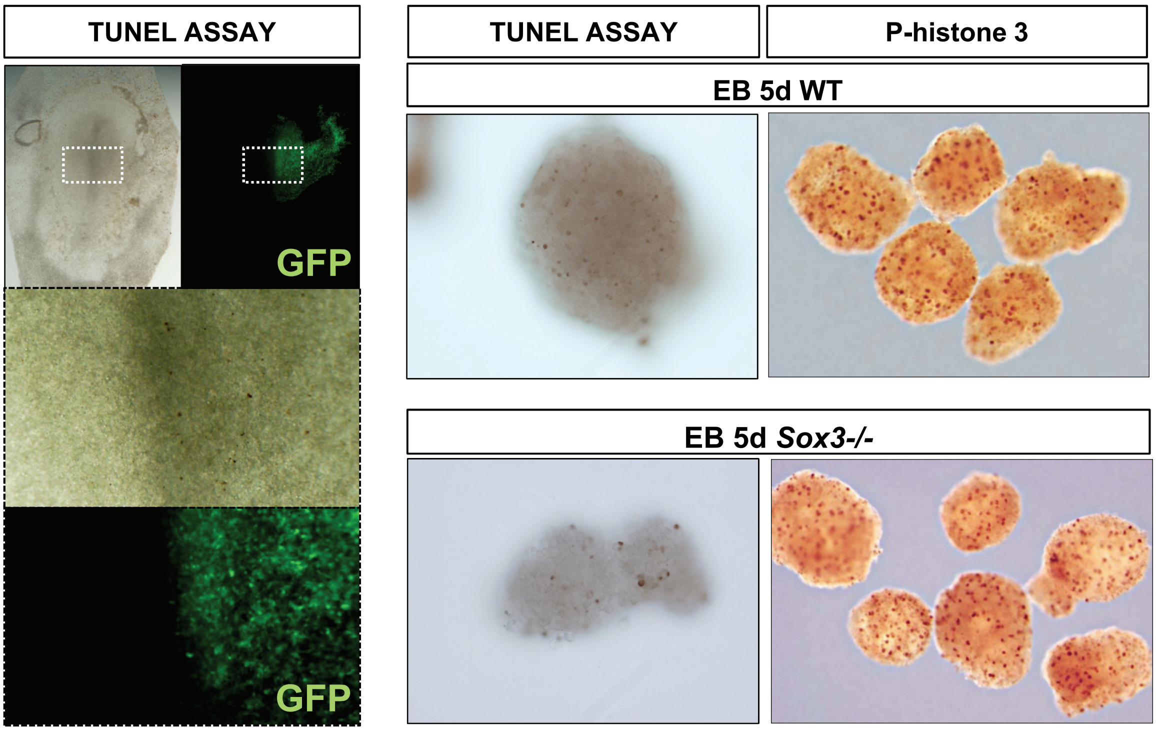

Supplementary Figure 2

Cell death and proliferation. Electroporation in chick embryos does not induce significant cell death as assessed by TUNEL staining (brown). The number of dead cells was less than 1% of the electroporated cells. The amount of dead cells detected is similar in wild type or *Sox3*-/- embryoid bodies (EB). Similarly, analysis of mitotic cells by phospho-histone 3 staining indicates that proliferation is not affected in *Sox3*-/- EB. Quantification of mitotic cells indicated that the difference between wild type (100%) and mutant embryoid bodies was less than 2% (98,6%).

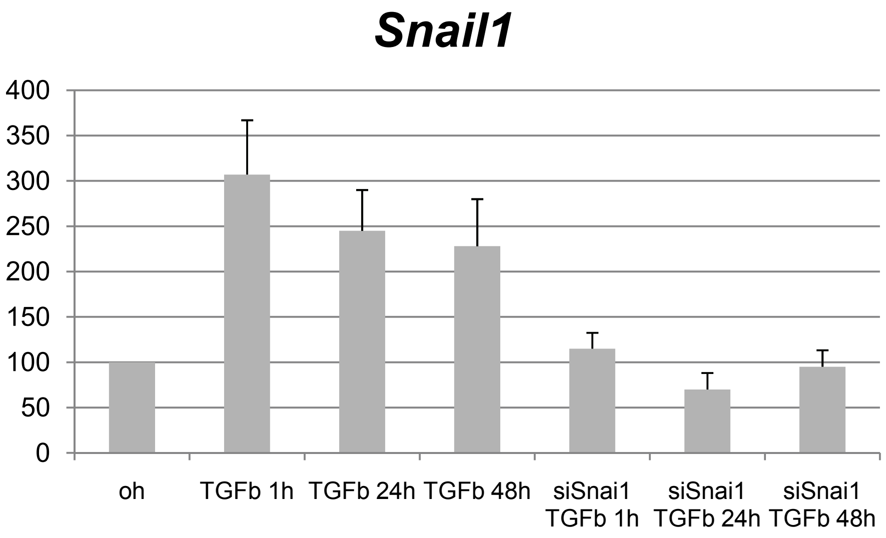

Supplementary Figure 3

Snail silencing by specific siRNAs. MCF7 cells were treated with TGFβ (2ng/ml) for 1h, 24h and 48h in presence or absence of specific siRNAs against *Snail1*. TGFβ-mediate Snail1 induction was efficiently blocked by Snail siRNA.

Supplementary Table 1: Primers sequences
(FW: forward; RV: reverse; Gg, chicken; Mm, mouse; Hs, human)

**Primer name**	**5' → 3' sequence**
KpnI Prom *Gg*Sox3 -5319 FW	ATGGTACCTTGGTGTGCAGTGGAA
KpnI Prom *Gg*Sox3 -4989 FW	GGTACCGGCAGGCTTCCTATTGTCTG
MluI Prom *Gg*Sox3 +1 RV	ATACGCGTCAGCTCGGGGAAAGGA
MluI Prom *Gg*Snai2 -1536 FW	ACGCGTTCTGCACTTGTTCTGGCATC
KpnI Prom *Gg*Snai2 -1473 FW	GGTACCGTGACGTGTCTTAACA
MluI Prom *Gg*Snai2 +1 RV	ACGCGTCATTTTGAAGGCAGGCTTTCT
*Gg*Sox3 FW	AGCGCTGCCCGCGGGAACCT
*Gg*Sox3 RV	AACAAAACAAACAAACAAAAAATTAAA
EcoR1*Gg*DNSox3 FW	GAATTCACCATGGATTACAAATACCGGCCCCG
EcoR1*Gg*DNSox3 RV	GAATTCTCATCCCGGCGCCCTGGTAGTG
EcoRI *Hs*SOX3 FW	GAATTCACCATGCGACCTGTTCGAGAG
EcoRI *Hs*SOX3 RV	GAATTCTCAGATGTGGGTCAGCGG
*Mm*Sox3 FW	GCCTGCTGGAGACTGAACTC
*Mm*Sox3 RV	GGTGGCAGGTACATGCTGAT
QPCR Prom *Gg*Sox3 SnailRE FW	TGCTTGGTGCAACAGAGTTT
QPCR Prom *Gg*Sox3 SnailRE RV	CCATGGAGCTTCTTGCTTTT
QPCR Prom *Gg*Snai2 SoxRE FW	TCTGCACTTGTTCTGGCATC
QPCR Prom *Gg*Snai2 SoxRE RV	AAAAGCCCCTCTGGTTCTGT
QPCR *Hs*ADAM12 FW	CAACGGGAAAGCAAAGAACT
QPCR *Hs*ADAM12 RV	GCGAGGGAGACATCAGTACC
QPCR *Hs*FN FW	GGTCTCCTGGGTCTCAGCTT
QPCR *Hs*FN RV	AGTGGCTGTGCTTGGAAGAT
QPCR *Hs*CLDN1 FW	CCGTTGGCATGAAGTGTATG
QPCR *Hs*CLDN1 RV	AGCCAGACCTGCAAGAAGAA
QPCR *Hs*E-CADH FW	TCATGAGTGTCCCCCGGTAT
QPCR *Hs*E-CADH RV	CAGCCGCTTTCAGATTTTCAT
QPCR *Hs*Snail1 FW	GCTGCAGGACTCTAATCCAGAGTT
QPCR *Hs*Snail1 RV	GACAGAGTCCCAGATGAGCATTG
QPCR *Hs*Snail2 FW	CCCTGAAGATGCATATTCGGAC
QPCR *Hs*Snail2 RV	CTTCTCCCCCGTGTGAGTTCTA
QPCR *Hs*Sox3 FW	AGACCAGGACCGTGTGAAAC
QPCR *Hs*Sox3 RV	AATTGTGCATCTTGGGGTTC
QPCR *Hs*36B4 FW	GTGATGTGCAGCTGATCAAGACT
QPCR *Hs*36B4 RV	GAAGACCAGCCCAAAGGAGA
QPCR *Mm*Snail1 FW	CCACACTGGTGAGAAGCCATTC
QPCR *Mm*Snail1 RV	TCTTCACATCCGAGTGGGTTT
QPCR *Mm*E-Cad FW	ACCTCCGTGATGAAGGTCTC
QPCR *Mm*E-Cad RV	CCGGTGTCCCTATTGACAGT
QPCR *Mm*actin FW	GGCACCACACCTTCTACAATG
QPCR *Mm*actin RV	GTGGTGGTGAAGCTGTAGCC
QPCR *Mm*Sox3	PPM04751A-200 from Superarray

,
